# Impact of organic carbon acquisition on growth and functional biomolecule production in diatoms

**DOI:** 10.1186/s12934-021-01627-x

**Published:** 2021-07-15

**Authors:** Thomas Kiran Marella, Raya Bhattacharjya, Archana Tiwari

**Affiliations:** 1 Algae Biomass and Energy System R&D Center (ABES), University of Tsukuba, Tennodai 1-1-1, Tsukuba, Ibaraki 305-8572 Japan; 2 Diatom Research Laboratory, Amity Institute of Biotechnology, Amity University, Noida, Uttar Pradesh India

**Keywords:** Diatoms, Carbon acquisition, Fucoxanthin, Mixotrophy, Heterotrophy

## Abstract

Diatoms are unicellular photosynthetic protists which constitute one of the most successful microalgae contributing enormously to global primary productivity and nutrient cycles in marine and freshwater habitats. Though they possess the ability to biosynthesize high value compounds like eicosatetraenoic acid (EPA), fucoxanthin (Fx) and chrysolaminarin (Chrl) the major bottle neck in commercialization is their inability to attain high density growth. However, their unique potential of acquiring diverse carbon sources via varied mechanisms enables them to adapt and grow under phototrophic, mixotrophic as well as heterotrophic modes. Growth on organic carbon substrates promotes higher biomass, lipid, and carbohydrate productivity, which further triggers the yield of various biomolecules. Since, the current mass culture practices primarily employ open pond and tubular photobioreactors for phototrophic growth, they become cost intensive and economically non-viable. Therefore, in this review we attempt to explore and compare the mechanisms involved in organic carbon acquisition in diatoms and its implications on mixotrophic and heterotrophic growth and biomolecule production and validate how these strategies could pave a way for future exploration and establishment of sustainable diatom biorefineries for novel biomolecules.

## Background

Diatoms are bacillariophytes which possess silicified cell walls and are one of the most dynamic class of photosynthetic primary producers of world’s oceans with around 30,000 to 100,000 species inhabiting both freshwater and marine habitats [1]. Due to their ability to thrive under varying physicochemical conditions like growing under the sea ice to thermal springs, they are ubiquitously found microalgae [2]. Besides being responsible for almost 40% oceanic and 20% global net primary productivity [3] diatoms are known to be evolutionarily outstanding because of their unique serial symbiotic events which makes their genomes chimeric with genes originating from prokaryotes, red algae, as well as green algae [4]. This symbiosis has equipped them with metabolic capabilities which are strikingly different from other algae class [5]. Diatoms processes silica bioaccumulation mechanism to build their intricately designed cell walls called frustules, which has substantial biomedical application in the form of an efficient drug delivery system [6]. In addition to this, they have the complete urea cycle and Entner-Doudoroff glycolytic pathway. They are known to store carbon as chrysolaminarin in vacuole [7] and possess a unique pigment signature with chlorophyll c and fucoxanthin as main light harvesting complex instead of chlorophyll b as other plants [8].

Diatoms also harbor a range of bioactive compounds, which are nutritionally very valuable for sustaining both mankind and the aquatic life. This includes polyunsaturated fatty acids like eicosapentaenoic acid (EPA) and arachidonic acid (ARA), phenolic compounds, terpenes, alkaloids, aldehydes, light harvesting pigments fucoxanthin (Fx), storage polysaccharide chrysolaminarin (Chrl) and numerous plant sterols [9]. They tend to accumulate up to 30–60% of the soluble carbohydrate in the form of Chrl, 1–6% Fx and 1–6% EPA on dry cell weight (DCW) basis [10]. These diatoms derived functional biomolecules in turn finds tremendous applications as anti-angiogenic, anti-obesity, antioxidants, antimicrobial, anti-inflammatory, and neuroprotective agents [11]. Studies report that monoacyl glycerides, oxylipins, fucoxanthin, fatty alcohol ester (nonyl 8-acetoxy-6-methyloctanoate, NAMO), stigmasterol and marennine are few of the compounds with significant anti-cancerous activity derived from diatoms [12].

Evolutionary transitions to acquire energy from different trophic modes is one of the key drivers of the dynamic metabolic adaptability in diatoms [13]. Diatoms contain physiological and metabolic machinery to couple respiration and photosynthesis by shuffling different enzymes and substrates between chloroplasts and mitochondria [14]. They have the potential to reduce a wide variety of organic carbon sources like glucose, glycerol, fructose, and acetate at different concentrations and under varying cultivation strategies for catabolism [10]. A myriad of environmental cues such as light intensity, color, salinity, and nutrient concentrations have a proportional impact on both the growth and ability of the alga to produce the bioactive compounds [15]. Hence in order to increase the competence of the diatom strains varied multi strategic approaches has been employed by researchers some of which includes increasing Fx content in *Cylindrotheca closterium* by inducing oxidative stress [16], or by exposing strains like *Pheodactylum tricornutm, Cylindrotheca fusiformis*, and *Coscinodiscus granii* to varying nitrogen concentration and light regimes respectively [17], or by supplementing the synthetic media with high silica content (3.0mM) and blue light for mass production in *P. tricornutum* [18]. Lately, few multi-omics based genetic approaches has also been applied for either overexpressing or manipulating the candidate genes of biomolecule biosynthesis [19, 20]. Nevertheless, experimenting by implementing chemical engineering by either modifying cultivation strategy, culture parameters or by optimizing the extraction procedure are more promising than most of the aforementioned methods. This has been recommended by the pioneering studies of Guo et al. [21], wherein *Cyclotella cryptica*, cultivated under both phototrophic and heterotrophic conditions. Several recent investigations on other microalgae classes like *Chromochloris zofingiensis, Galdieria* sp., *Auxenochlorella protothecoides, Scenedesmus obliquus* and *Scenedesmus bijuga* have also reported high adaptability of growing under mixotrophic mode and how efficient organic carbon conversion rate has significantly upgraded the biomass production which in turn has been utilized for biodiesel production and wastewater recycling [22–24]. However, since metabolic pathways differ between different algae classes significant optimization of diatom cultivation parameters is quintessential for understanding the interplay of carbon acquisition, partition and regulation to achieve higher biomass and value-added product productivity [24].

**Fig. 1:**
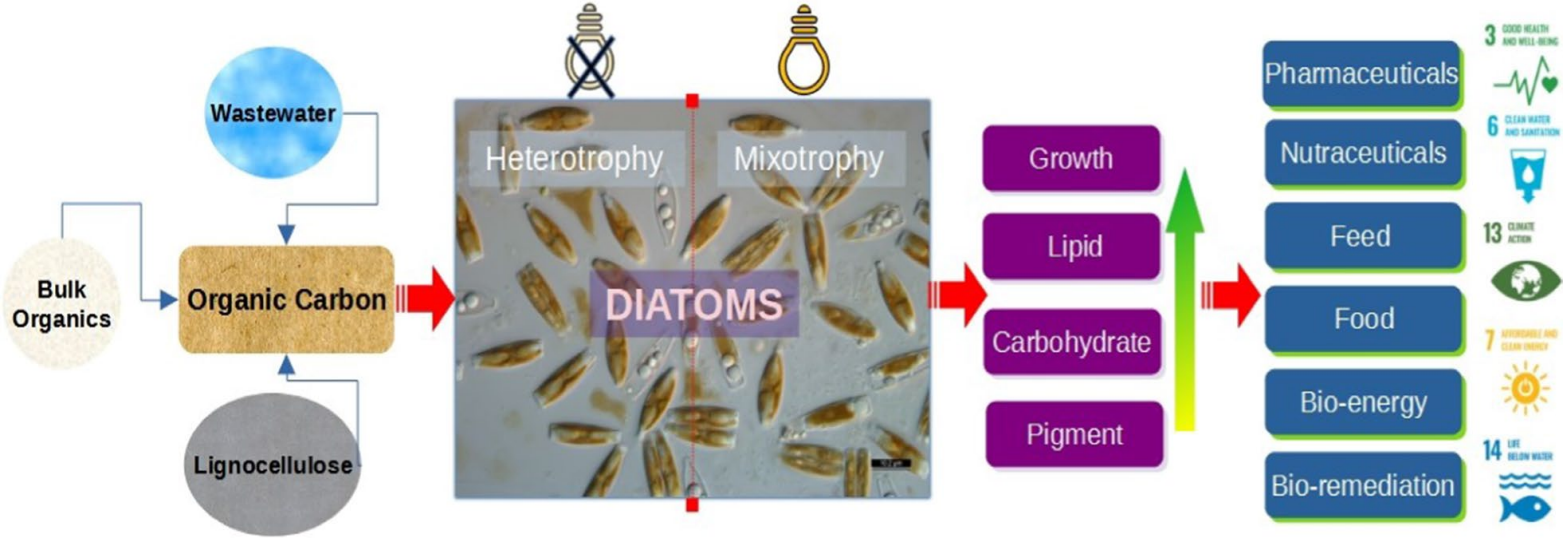
Schematic overview of impact of organic carbon on diatoms and its implications on various value-added product generation and related sustainable development goals

This review aims to highlight the influence of diverse organic carbon sources in triggering the diatom growth and biomolecule productivity facilitating their commercial applications (Fig. 1). Since the robustness of the metabolic networks depend on the evolutionary complexity of the strain, niche-specific differences in diatom species could be identified by strategic cultivation approaches Thus, to circumvent the limitations of genetic engineering and enhance high value added biomolecule production in a sustainable way by utilizing cost-effective industrial co-products primarily organic carbon compounds, the metabolic versatility of diatoms can be understood by exploring its adaptability to different nutrition modes, namely, photo trophy, mixotrophy and heterotrophy.

### Mechanisms involved in organic carbon assimilation in diatoms

Diatom algae are products of a unique secondary endosymbiotic event between heterotrophic eukaryote and a red alga. This event resulted in amalgamation of genes and resultant metabolic pathways which are unique to diatoms like complete urea cycle, carbon concentrating mechanisms (CCM), four layered chloroplasts, C4 photosynthesis etc. [25–27]. In diatoms, majority species prefer photo-autotrophy as the preferred carbon acquisition mode which converts CO_2_ in the presence of light to reduces carbon. This process is mainly performed though Rubisco and Calvin cycle in mitochondria where photosystem I and photosystem II are utilized to generate NADPH and ATP [28]. In heterotrophy, glucose was converted to glucose 6 phosphate (G6P), which can be readily used for multiple purposes like carbon storage, respiration and cell organelle synthesis depending on cellular requirement. Heterotrophic algae perform carbon acquisition by three main central pathways namely glycolysis, tricarboxylic acid cycle and oxidative pentose phosphate pathway (PPP) [29]. In diatoms pyruvate is the key metabolite from glycolysis which is used to biosynthesize amino acids, lactate, ethanol, and acetyl CoA (ACCoA). ACCoA is the major precursor for fatty acid synthesis, and it is also further processed for ATP generation through Krebs cycle. Unlike glycolysis PPP is predominantly anabolic resulting in generation of NADPH. Carbon assimilation and storage in diatoms in mainly controlled by both glycolysis and gluconeogenesis, when cells carbon needs were met, pyruvate is converted to storage carbon which is chrysolaminarin. Unlike other algae classes in diatoms central carbon metabolism is not only controlled by mass balance but also partially controlled by compartmentalization pathways between different cell organelles, like in photosynthetic diatoms oxidative phosphorylation reaction of PPP was confined to cytosol whereas in non-photosynthetic diatoms PPP is in plastid [13].

**Fig. 2:**
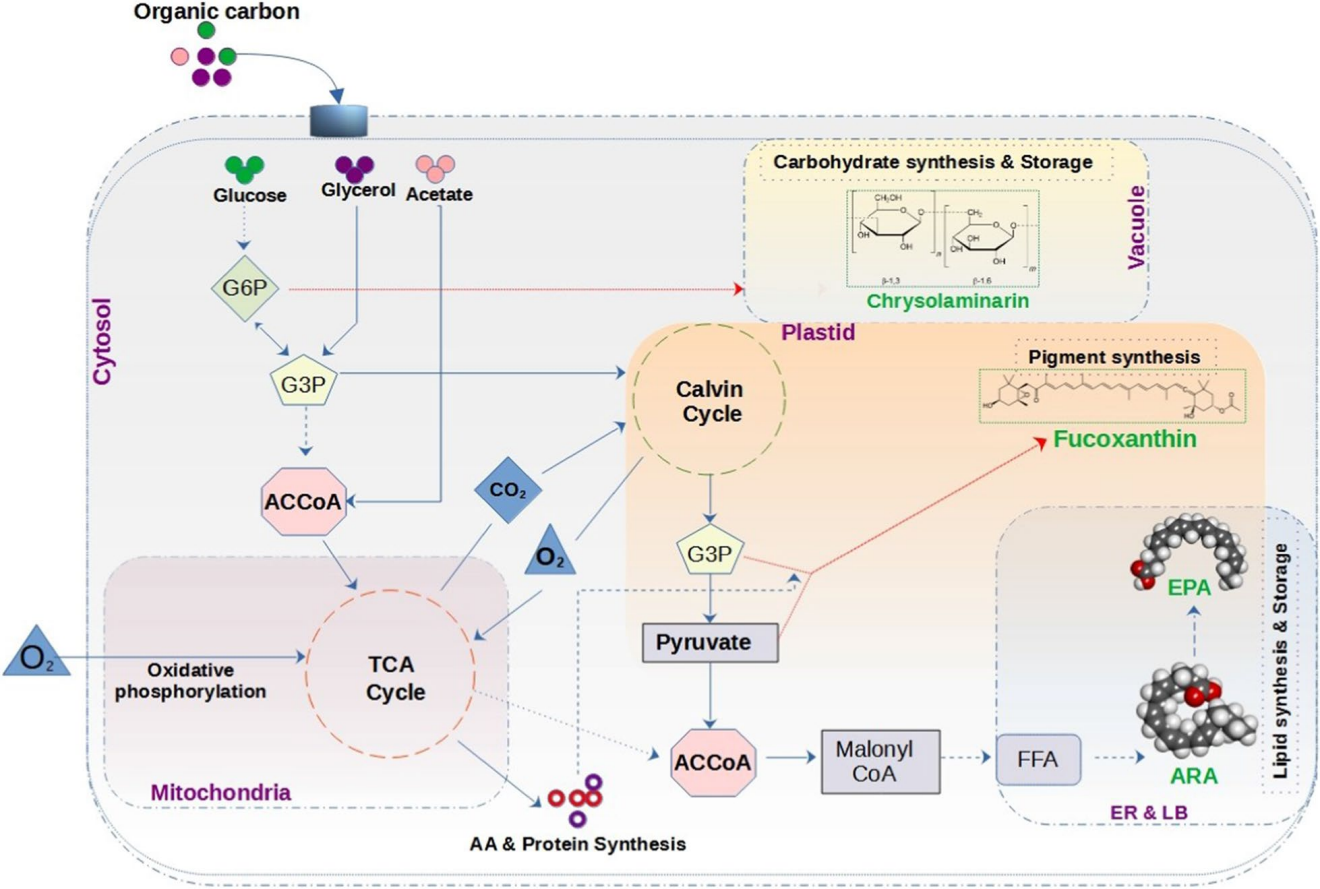
Scheme of pathways involved in organic carbon assimilation and its conversion to functional molecules during heterotrophic and mixotrophic growth in diatoms. Compound abbreviations are. G6P- glyceraldehyde-6-phosphate; G3P- glyceraldehyde-3-phosphate; ACCoA- Acetyl-CoA; TCA cycle-Tricaboxylic acid cycle; FFA; free fatty acids; ER-Endoplasmic reticulum; LB-Lipid bodies

In diatoms glucose is most preferred carbon substrate as in other algae groups due to its higher reducing power compared to other substrates [30]. In diatom *N. alba* dry cell weight of 500 mg per gram of glucose was achieved in fermenter cultures which is higher than other organic carbon sources tested [31]. But other commercially important species like *Pheodactylum* sp. and *Cylindrotheca* sp. which are predominantly mixotrophs and cannot utilize glucose in dark instead they prefer glycerol and acetate as carbon source [32, 33]. In diatoms along with glucose glycerol and acetate are also reported as preferred organic carbon sources which can sustain high growth rate. Glucose assimilation in heterotrophic mode is mainly carried out by pentose phosphate pathway (PP) while in mixotrophic mode Embden-Meyerhof-Parnas pathway (EMP) is used for glycolysis. Glucose acquisition involves an added intermediate step to produce G3P which is the main precursor for ACCoA, but glycerol can be directly converted to G3P whereas acetate can directly be incorporated as ACCoA (Fig. 2). In cyanobacteria carbon assimilation gene expression pattern showed no significant difference between all three modes of cultivation but reductive EMP pathway genes from glycogenesis and Calvin cycle was upregulated during mixotrophy and photo-autotrophy [34]. This indicated the carbon pathway is not only regulated at gene level but also on mass balance although similar studies were not done on diatom species but studies on non-photosynthetic diatoms have shown that carbon assimilation pathways are governed at transcript level and on substrate concentration [13].

Acetate can support diatoms in both mixotrophic and heterotrophic growth [35]. The main advantage of using acetate as carbon substrate is single step conversion of acetate to ACCoA by acetyl CoA synthase [36]. In diatoms this process was performed through glyoxalate pathway in plastids and by TCA cycle in mitochondria. Two of the key enzymes involved in glyoxylate cycle, malate synthetase and isocitrate lyase both these enzymes were found to be localized to mitochondria in diatoms [29]. Glycerol is increasingly used in heterotrophic and mixotrophic nutrition of microalgae due to its increased availability by being a by-product of biodiesel industry. It is known to be physiologically compatible organic carbon source due to its effect on cell membranes and enzyme machinery even when used in high concentration [37]. Besides acting as the backbone for TAG synthesis, glycerol is either phosphorylated using ATP by glycerol kinase into glycerol-3- phosphate (G3P) or is degraded by glycerol-3-phosphate dehydrogenase into dihydroxyacetone phosphate (DHAP). Finally, it enters the Embden-Meyerhof-Parnas (EMP) pathway as G3P or DHAP and is further converted into pyruvate which then regulates the central carbon metabolism through the TCA cycle. In *Pheodactylum tricornutum* glycerol uptake was upregulated in mixotrophic growth compared to heterotrophy. Subsequently, glycerol also induced significant variations in flux in reactions associated with the carbon cycle pathways like Calvin-Benson cycle, TCA cycle, PPP, photorespiration, and glycolysis [38]. Glycerol uptake increased pyruvate content which indicates that glycerol can induce higher respiratory activity in diatoms. In *P. tricornutum* glycerol influenced carbon uptake and storage rate by regulating downstream part of glycolytic pathway which included acetyl-CoA and TAG production and upstream of gluconeogenesis which influenced carbohydrate production. In green algae species like *Chlorella* glycerol concentration influenced growth and storage compound concentration and in certain species high glycerol concentration had negative impact on growth but in diatom algae mixotrophic growth on glycerol even at high substrate concentration (10–20 g L^− 1^) promoted higher biomass and lipid productivity [38, 39]. In recent genomic studies on non-photosynthetic diatom *Nitzschia* sp. which can utilize unusual complex carbon sources revealed presence of β-ketoadipate pathway which catalyzes the cleavage of benzene rings to form carboxymuconic acid which can be utilized as carbon source [13]. Though this pathway of β-ketoadipate was previously found only in eubacteria and fungi [40], its presence in diatoms too further validates its ability to utilize complex organic carbon substrates. During heterotrophic and mixotrophic growth of microalgae on organic carbon sources cellular respiration plays a key role in ATP generation through oxidative phosphorylation. In diatoms respiration plays an important role as energy source and other key cellular processes which includes lipid and PUFA production. During high density culturing in fermenters dissolved oxygen is the major limiting factor influencing biomass and EPA production is found to be a major substrate for EPA synthesis [41].

In diatoms, chrysolaminarin is the major storage polysaccharide and its biosynthetic pathway from organic carbon sources was recently elucidated [42]. Organic carbon source like glucose is converted to G6P and then to G1P by action of enzyme phosphoglucomutase (PGM). This is the initial step involved in glucose assimilation whereas glycerol is directly converted to G3P. Enzyme uridine-di-phosphate-glucose-pyrophosphokinase (UPP) converts G1P to UDP glucose from which 1,3-β glucose was synthesized by the action of 1,3-β glucan synthase (BGS1). The final biosynthesis of Chrl occurs in vacuole where 1,3-β glucose was used as substrate to form Chrl by enzyme 1,6-β transglucosylase. Fucoxanthin which is the main light harvesting pigment in diatoms is synthesized through both mevalonic acid (MVA) and methylerythritol phosphate (MEP) pathways in auto heterotrophic and heterotrophic cultivation modes. The principal substrate for Fx synthesis is G3P, which is synthesized from glucose, glycerol and from pyruvate though ACCoA from TCA cycle (Fig. 2). G3P is converted to isopentenyl pyrophosphate (IPP) which further forms phytoene by catalysis of phytoene synthase (PSY). Phytoene through a series of desaturation reactions by phytoene desaturase (PDS) forms β-carotene. β-carotene by hydroxylation and epoxidation to form zeaxanthin and violaxanthin respectively [43]. Violaxanthin is converted to neoxanthin, although molecular mechanism of synthesis of Fx and Dx from neoxanthin is not yet clear in diatoms, but recent studies found that ketolation and acetylation reactions are involved in Fx synthesis [43]. Diatom lipids are rich in LC-PUFA’s like EPA, DHA and ARA. ACCoA acts as the major building block for fatty acid synthesis by acting as substrate for acyl chain elongation. During mixotrophic and heterotrophic growth ACCoA is sourced from mitochondria through TCA cycle and from plastid by Calvin cycle. Through action of ACCoA-carboxylase (ACCase) enzyme malonyl-CoA is formed which is further transformed to malonyl acyl carrier protein (ACP) by malonyl-CoA:ACP transacylase (MAT). Acyl Chain elongation from ACP was catalyzed by ketoacyl-ACP synthase (KAS) to form individual fatty acids. In diatoms LC-PUFA formation occurs through C18:1 which is synthesized in plastid and transferred to endoplasmic reticulum. A series of desaturation and elongation reactions catalyzed by fatty acid desaturase (FAD) and fatty acid elongase (FAE) respectively facilitates formation of EPA and DHA from 18:1-CoA [44].

### **Traditional diatom cultivation systems and their limitations**

The mode of cultivation in paramount for attaining desired productivity in industrial scale production of microalgae. They can be cultivated mainly by four different modes: heterotrophy, wherein organic carbon acts as the sole carbon source, mixotrophy in which both light and organic carbon acts as C source, photoheterotrophy in which organic carbon is utilized in presence of light as main energy source, and phototrophy where inorganic carbon mainly CO_2_ is utilized in presence of light [45]. In diatoms, of the two commonly used systems, though outdoor open pond system promotes more growth, unregulated light availability that is light with higher intensity can lead to photooxidation and that with less intensity can hinder photosynthetic efficiency. phototrophic open pond systems result in low biomass productivity not greater than 0.5 g L^− 1^ [46]. Due to less biomass density the amount of liquid which needs to be processed to obtain biomass is extremely high which increases overall downstream processing costs. Open pond systems also prone to protozoal and competing algae contamination which makes single cell culturing labor and cost intensive. These limitations make open pond cultivation unsustainable for large scale biomolecule production [47]. PBR based cultivation for diatoms use flat panel, stirred tank, tubular, and biofilm-based culture systems. Main advantage of employing tubular photobioreactors is their energy efficiency but limitation of this technique was upscaling cost [48]. Stirred tank systems are like fermenters but providing light and maintaining controlled conditions makes it expensive especially for phototrophic strains. Attached cultivation systems like algae biofilm reactors are innovative additions to the existing traditional systems but not yet proven for large scale production as it needs the diatom strain to be cultivated to be able to grow on a solid substrate as an attached biofilm. Although benthic diatoms like *C. closterium* and *C. fusiformis* can form biofilms in their natural habitats, but they are not tested yet in these biofilm cultivation systems [49]. Due to these pertinent limitations growing diatoms in mixotrophic and heterotrophic modes may be an alternative to attain high biomass productivity which is a prerequisite for industrial scale applications.

### Cultivation modes of diatoms

In heterotrophy microalgae utilize external inorganic carbon source through an aerobic process catalyzed by oxygen dependent oxidative phosphorylation. Mixotrophic growth is triggered by simultaneous breakdown of both inorganic and organic carbon sources with light as energy source in which photosynthetically fixed CO_2_ acts as primary inorganic C source. Diatoms are well adapted to both heterotrophic and mixotrophic growth using mono, poly and complex organic sources [50]. Diatoms are believed to contain essential mechanisms to perform both phototrophic and heterotrophic growth especially under light limited conditions [51]. This ability gives them advantage to out compete other algae groups especially in benthic environments where light is limited [52]. Diatoms are known to assimilate multiple exogenous carbon sources like glucose, amino acids, glycerol, acetate, lactate, ethanol, saturated fatty acids, and pyruvates [53]. Out of 10,000 reported diatom species a small subgroup of diatoms exclusively belonging to pennate *Nitzschia* sp. are known to be apochlorotic species which has lost required machinery for photosynthesis like reduced plastids and thylakoids with reduces photosynthetic related genes and complete absence of photosynthetic pigments, in spite of their small number their growth rates in 3–4 times higher than their photosynthetic cousins which makes them ideal candidates for high density culturing [54].

Many commercially cultivated diatom species are well adapted to grow under high agitation due to their innate ability to sustain viability under shear forces encountered in rough seas, this ability makes them ideal candidates for high density growth in fermenters. Species like *N. alba* and *C. meneghiniana* can grow heterotrophically on glucose more rapidly than in autotrophy [55]. In model diatom *Pheodactylum* mixotrophic growth on glycerol induced substantial increase in growth, lipid, and carbohydrate productivity [38]. Although many diatom strains are cultured in mixotrophic and heterotrophic modes till now only four strains are successfully employed for high cell density culturing namely *Nitzschia alba* [56], *Nitzschia laevis* [57], *Cyclotell criptica* [46] in heterotrophic mode and *Pheodcatylum tricornutum* in mixotrophic mode [33].

### Advantages and limitations of heterotrophy and mixotrophy

In mass cultures compared to photo-autotrophic growth heterotrophic and mixotrophic growth of diatoms has advantages like increased growth rate and biomass productivity, reduced respiratory biomass loss, higher lipid and EPA productivity, higher carbohydrate (Chrl) content, enhanced lipid content per DCW even under nutrient replete growth, reduced photochemical quenching, reduced light requirement without loss in productivity, increased pigment content (Fucoxanthin) and less bacterial, copepods and invasive algae contamination [58, 59]. Heterotopic diatoms can be grown in industrial scale fermenters which are traditionally used for growing bacteria and yeast and the cost of ush fermenters and existing knowledge in maintaining controlled conditions in them can be easily adapted to grow diatoms with short seed time. Biomass density of 40 g L^− 1^ and productivity of 15–20 g L^− 1^ d^− 1^ of diatom, *Nitzschia alba* cultivated in fermenter cultures, is recorded as the highest values of density and productivity reported in diatoms so far [46]. Since such species are oddly adapted to heterotrophic modes, culturing and maintaining their monocultures under these conditions eases as well as enhances production of high value chemicals for pharmaceutical and nutraceutical sectors. In model diatom *Pheodactylum* photo-autotrophic cultivation in photobioreactors is affected by severe light limitation due to mutual shading effect of cells which resulted in decreased productivity [33]. Further due to low cell density downstream processing steps which involves biomass harvesting needs higher energy which further creates a cascading effect of longer processing times and escalation of production costs [60]. So, in commercial process use of heterotrophy or mixotrophy can lead to reduction in culturing time and downstream processing costs.

Major limitation of growing microalgae in heterotrophic mode is cost of carbon substrates mainly glucose [61]. Due to its absolute requirement and its direct influence on biomass content it is an irreplaceable commodity. So, screening for diatom species which uses other cheap carbon sources like lignocellulose or grow directly on non-reducing sugar wastes with no pretreatment is needed to reduce the production cost. Due to increased availability of wastewater from industrial, municipal, and agricultural sources which are rich in organic carbon content and other major nutrients essential for diatom growth use of such cheap nutrient sources can lead not only to generate valuable biomass but also in bioremediation of excess nutrients which are major cause of cultural eutrophication in urban and peri urban areas around the world [62]. Other limitations include high cost involved in use of fermenters and their maintenance especially for low-cost bye products like for biofuel production [63].

### Organic carbon substrates on diatom growth

**Fig. 3:**
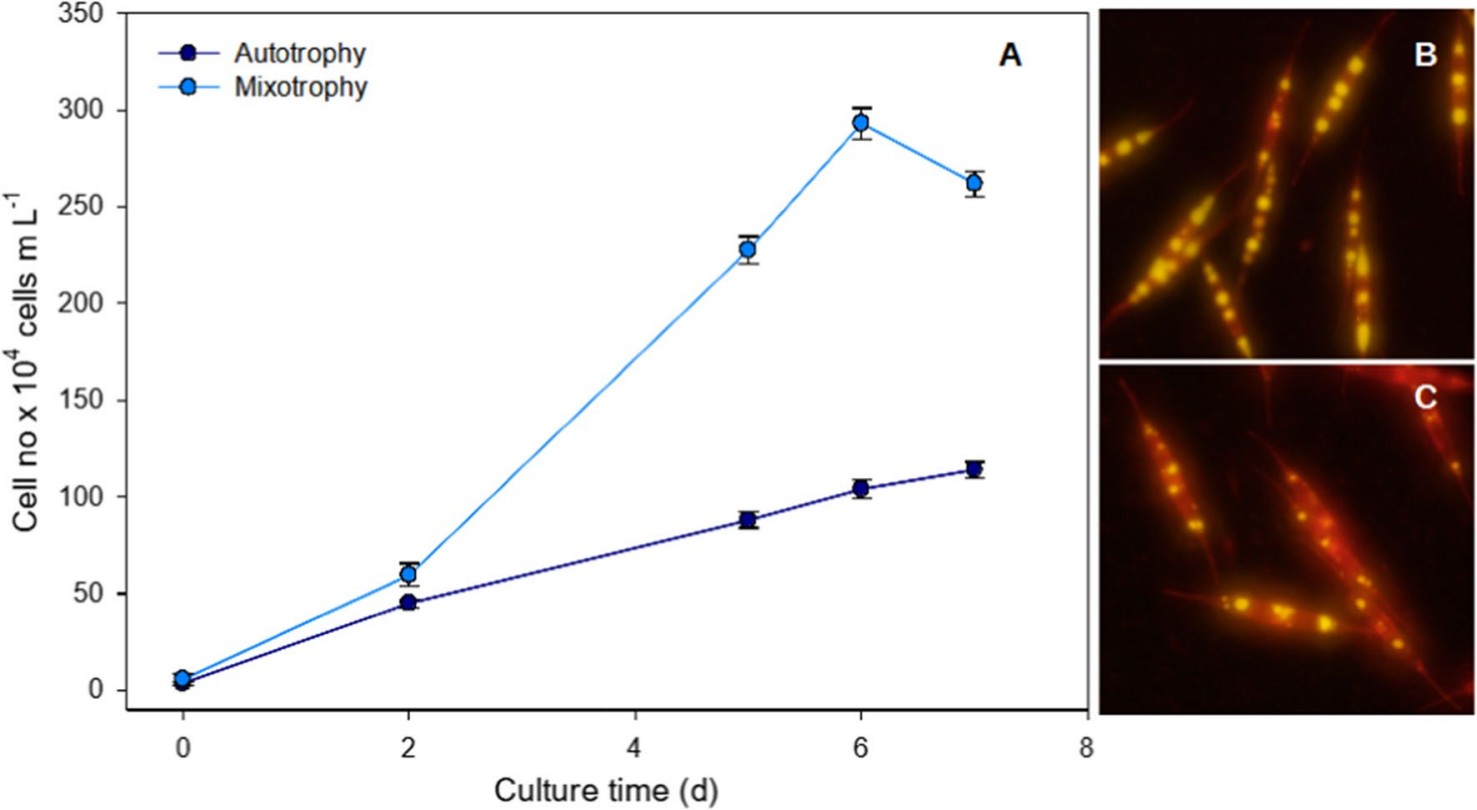
Comparison of growth and lipid production between photo-autotrophic and mixotrophic growth in *Cylindrotheca* sp. Growth curve of of cultures under different modes (**A**). Difference in lipid content at stationary phase of growth visualized by nile red staining in cells grown in Mixotrophic (**B**) and photoautotrophic mode (**C**)

Though research on optimizing cultivation conditions of diatoms has long been one of the most preferred areas of interest in microbial studies, but their growth on organic carbon in dark was conclusively established by Lewin [50] while working with pennate diatom cultures. He reported the growth and effective utilization of carbon sources like glucose, fructose, glycerol, and acetate under heterotrophic conditions. Thereafter they showcased that diatom like *Nitzschia alba, Cylindrotheca fusiformis, Nitzschia angularis* var. *affinis*, *Nitzschia laevis* could grow heterotrophically, evidently, using lactate, succinate, glutamate as well as glucose [51, 64] with enhanced growth rate. Later, Tan and Johns [58] screened different *Nitzschia* strains for enhancing both heterotrophic growth and EPA production. Since mixotrophic cultivation of microalgae had been reported to trigger higher growth rate than photoautotrophic mode as had been demonstrated by Hellebust [65], in *Cyclotella cryptica*, when grown on glucose, subsequent practices gave promising results with *Navicula saprophila* in presence of acetic acid [66]. Ever since then, researchers have been focusing on implementing both trophic modes to enhance growth and biomass productivity of diatoms to comprehend the increasing need of natural source of fuel, food, and pharmaceuticals. Mixotrophic conditions could also increase respiration rate besides stimulating higher biomass concentration in *Phaeodactylum tricornutum* [33]. With the complete genome being sequenced, this model diatom has been exhaustively exploited for studying such adaptive cultivation practices over the years [67]. This in turn has also enabled the researchers not just to demonstrate but also investigate the key metabolic changes and pathways being targeted when an obligate photoautotroph is driven under varied environmental conditions. Mus et al. [68] had reported both enhanced growth and lipid productivity in mixotrophically grown *P.tricornutum* under carbon sources like glucose, starch and acetate. Glucose utilization and the metabolism was initially studied by isotopic engineering for unravelling the pathways and interactions [30]. As this cellular crosstalk because of mixotrophy was central for understanding underlying metabolism, coupling metabolic modelling with such practices thereafter had paved new arenas for diatom research. Pioneering work in this had been done by Villanova et al. [38], wherein they deployed transcriptomics, metabolomics as well as lipidomics approaches for revealing the role of glycerol transporter in growth and TAG accumulation. However, besides *P.tricornutum*, and the obligate and facultative heterotrophs like *Nitzschia alba* and *Cyclotella cryptica* several other diatoms like *Synedra acus, Nitzschia laevis, Cylindrotheca Closterium*, and *Cylindrotheca fusiformis* have also been reported to sustain as well as produce significant amounts of biomass when grown either hetero- or mixotrophically (Fig. 3). A detailed account of different organic substrates and its influence on mixotrophic and heterotrophic growth on diatoms was summarized in Table 1. These microalgae have shown that besides growth, changing the trophic mode of nutrition could also manipulate either the lipid or the fucoxanthin content drastically. Recently, a mixotrophic grown report of *Thalassiosira pseudonana*, on crude glycerol at 2.5 g L^− 1^ concentration, has also evidently revealed an increase in biomass productivity as well as in lipid productivity and accumulation of short chain fatty acids [69].

**Table 1:** Effect of different cultivation modes on biomass and functional biomolecule production in diatoms

Diatom species	Culture Mode	Cultivation techniques	Carbon substrate (optimum concentration)/ growth conditions	Growth rate/Biomass concentration	Functional biomolecule	References
*Cyclotella cryptica*	Mixotrophic	Batch	Glucose (10 g L^− 1^ )	Biomass- 1.72 g L^− 1^	Fucoxanthin- 3.38 mg L^− 1^d^− 1^; 1.29 % DCW	[21]
*Cyclotella cryptica*	Autotrophy	Batch; two stage	Light 200 µE; L:D 14:10 h; High nutrient medium + CO_2_	Biomass 1.25 g L^− 1^	Higher biomass and Lipid productivity	[78]
*Cylindrotheca fusiformis*	Heterotrophic	Batch	Lactate; Succinate (0.045 M)	Maximum doubling time was 33 and 30 h respectively under each condition	Comparable levels of Chlorophyll a, c; β carotene and fucoxanthin was detected as under light conditions; However, Diadinoxanthin could not be detected	[65]
*Cylindrotheca fusiformis*	Mixotrophy	Batch	Glycerol; Acetate	Biomass- 0.8 g L^− 1^	na^a^	[32]
	Autotrophy	Batch	*f*/2 + Nualgi (Nutrient mixture)	Biomass- 0.7 g L^− 1^	EPA 24 % of TFA	
*Navicula saprophila*	Mixotrophic	Batch	Acetic acid; Ethanol; Glucose (1mM)	na	EPA (acetate;14.3 mg g^− 1^, Ethanol;7.3 mg g^− 1 ,^ 10.4 mg g-^1^ Glucose)	[66]
*Nitzschia alba*	Heterotrophic	Batch	Lactate; Succinate; Glucose; Glutamate	Enhanced growth rate - (µ) 0.106	Enhanced EPA	[79]
*Nitzschia alba*	Heterotrophic	Fed-batch ; Fermentor	Glucose	30 g L^− 1^	Total lipid 60% DCW; EPA 2–4 % TFA	[56]
*Nitzschia angularis var. affinis*	Heterotrophic	Batch	Glutamate (0.003 to 2mM); Alanine + Glucose; glucose + Amino acids	High growth with Glucose + Glutamate	na	[80]
*Nitzschia brevirostris*	Heterotrophic Mixotrophic	Batch Batch	Glucose (0.1 M)	25.4 g L^− 1^ 35.1 g L^− 1^	EPA- 5.28 %; Fucoxanthin- 0.08 % of DCW EPA- 6.14 %; Fucoxanthin- 0.22 % of DCW	[81]
*Nitzschia laevis*	Heterotrophic Mixotrophic	Fed-batch; Two stage	Glucose (5 g L^− 1^ ); Varying light	Biomass 2.22 g L^− 1^ DCW Biomass 1.91 g L^− 1^ DCW	Fucoxanthin-0.89 % DCW Fucoxanthin at10µmol m^− 2^ s ^− 1^ -1.11 % DCW, 5.42 mg L^− 1^d^− 1^	[75]
*Nitzschia laevis*	Heterotrophic	Fed-batch; Fermenter	Acetate; Glucose	22.1 g L^− 1^	EPA- 0.70 g L^− 1^	[82]
*Nitzschia laevis*	Heterotrophic	Fed-batch; Fermenter	Glucose	40.0 g L^− 1^	EPA- 1.1 g L^− 1^	[83]
*Nitzschia laevis*	Heterotrophic	Batch	Glucose	na	EPA yield (17 mg g^− 1^ DCW)	[58]
*Nitzschia laevis*	Heterotrophic	Batch	Glutamate (0.16 to 2mM) and Glucose (1.2 mM)	Rapid growth rate; 48 h with glutamate and 24 h with glucose alone	na	[84]
*Phaeodactylum tricornutum*	Mixotrophic	Batch	Glycerol (100mM)	Specific growth rate (day^− 1^)-0.18; Biomass 173 mg L^− 1^	Significant increase in respiration rate	[85]
*Phaeodactylum tricornutum*	Mixotrophic	Batch	Glycerol (0.02 M)	Promoted growth	Enhanced photorespiration	[86]
*Phaeodactylum tricornutum*	Mixotrophic	Batch	Sodium acetate; Starch; Glucose (0.5 to 5 g L^− 1^)	Maximum biomass- 1.16 gL^− 1^	Low glucose content increased the total lipid content and productivity (0.29 g g ^− 1^ and 0.017 g^− l^ d^− 1^)	[87]
*Phaeodactylum tricornutum*	Mixotrophic	Fed-batch; PBR	Glycerol (0.1 M)	Biomass − 16.2 g L^− 1^ DCW ;17.5 mg L^− 1^ h^− 1^	EPA increase − 33.5 mg L^− 1^ d^− 1^	[33]
*Phaeodactylum tricornutum*	Mixotrophic	Batch; PBR	Enriched ESAW medium + Glycerol	Biomass – 5.3 g L^− 1^	EPA 3.98 mg L^− 1^ d^− 1^	
	Mixotrophy	Batch; PBR	Enriched ESAW medium + Glycerol + HCO_3_	Biomass – 11.55 g L^− 1^	EPA 9.51 mg L^− 1^ d^− 1^; Fx 1.97 mg L^− 1^ d^− 1^	[38]
	Autotrophy	Batch; PBR	Enriched ESAW medium + Glycerol	Biomass – 1.6 g L^− 1^	EPA 1.40 mg L^− 1^ d^− 1^	
*Synedra acus subsp. radians*	Mixotrophic	Batch	Glucose (40mM); Glycerol (80mM)	Promoted growth	Enhanced chrysolaminarin, EPA and DHA	[88]
*Thalassiosira pseudonana*	Mixotrophic	na	Crude glycerol (2.5 g L^− 1^)	Increased biomass- 0.75 g DW L^− 1^	Increase in short and long chain fatty acids (C14:0; C18:4(n-3))	[69]

^a^ Data not available

### Influence of the externally supplemented carbon substrates on bioactive compounds accumulation in diatom cells

Sustainable production of high value-added bioactive compounds like ω-3 fatty acids (EPA, DHA), chrysolaminarin, fucoxanthin, peptides, and sterols amongst others is one of the major challenges which requires enhanced production and accumulation of these metabolites by adapting modified cultivation parameters. Assimilated carbon forms the major building block for biosynthesis of major functional biomolecules likes EPA, Fx and Chrl and thus directly influences their overall productivity in diatoms. Effect of different culture modes and organic carbon substrates on lipid, EPA and fucoxanthin productivity was summarized in Table 1. Recent transcriptomic analysis of diatoms revealed the major pathways involved in biosynthesis for these compounds [70]. In diatoms, Acetyl CoA formed through multiple processes like beta oxidation of acyl CoA in cytosol, from citate metabolism through TCA cycle and from pyruvate through calvin cycle in plastid. Acetyl CoA after conversion to malonyl CoA in both cytosol and in plastid is converted through multiple steps to free fatty acids (FFA) which are then transported to endoplasmic reticulum (ER) which acts as major reaction center for PUFA and TAG synthesis in diatoms. In diatoms glycerol upregulated genes responsible for synthesis and regulation of key building blocks like pyruvate and acetyl-CoA which directly influenced lipid and carbohydrate production [38].

In *Pheodactylum* glycerol and nitrate supplementation enhanced TAG productivity even in nitrogen replete condition which is quite interesting as many microalgae including diatoms are known to accumulate lipids in response to N limitation [38]. Along with TAG glycerol also influenced EPA productivity which suggests that glycerol can induce metabolic changes which resemble N replete conditions this observation is quite important in the context of PUFA production from diatoms as many studies reported a decrease in EPA during late stationary phase compared to exponential phase indicating that EPA being the main structural phospholipid in cell membranes and polar glycerolipid is converted to MUFA like C18:1 and C16:1 which are predominantly storage lipids. Glycerol is also found to upregulate a key EPA biosynthesis enzyme, stearoyldesaturase (2.27-fold) [71]. So, to maintain high EPA content even during lipid accumulation phase use of glycerol as carbon source can be more ideal than use of two stage cultivation in N deplete medium.

Fatty acid synthesis in algae is initiated from carbon coupling from acetate and malonyl-CoA through acetyl-CoA derived from pyruvate by inorganic or organic carbon acquisition. To perform FA synthesis amount of reducing power through NADPH is the major limiting factor (16 molecules of NADPH per molecule of C18 FA synthesis). In dark growth PPP pathway provides more reducing power as NADPH compared to autotrophic conditions [72]. Under heterotrophic growth many green algae species reported increase in saturated fatty acid content but in diatoms EPA and DHA content is higher [46, 57]. But surprisingly obligate heterotrophic diatoms lack plastid PPP pathway, and it is still unclear how they can meet the demand for NADPH for FA synthesis [13]. Cellular respiration also plays a key role in enhancing EPA content in diatoms. In *N. alba* oxygen concentration in the medium directly influenced EPA productivity, with O_2_ concentration of 30% above air saturation resulting in higher EPA content [41]. Heterotrophic growth in diatoms resulted in two to three times higher ω-3 fatty acid productivity compared to autotrophic growth. This productivity is one to two orders higher than bacterial or fungal systems [56].

Along with EPA chysolaminarin which is a soluble carbohydrate β-1,3 glucan is the major storage form of carbohydrate in diatoms [73]. Chrl is mainly stored in vacuole, the principal pathway for Chrl synthesis in diatoms is conversion of G6P obtained from glucose to be converted to Chrl by enzyme phosphoglucomutase [74]. So, availability of carbon substrates directly influences Chrl content in diatoms. In *N. laevis* organic carbon supplementation increased biomass and EPA productivity by around 8- fold compared to autotrophic cultivation [57]. Cellular response of diatoms to type of carbon source in terms of lipid, EPA and Fx accumulation is species dependent. Comparatively glucose dependent growth has shown varied response in diatoms species studied, in *N. laevis* it increased EPA and Fx content whereas s in *Pheodactylum* it reduced Fx productivity significantly [10, 75]. Fucoxanthin biosynthesis pathway is diatoms is light dependent so heterotrophic growth has a negative impact on Fx content. But mixotrophic cultivation using glycerol in *Pheodactylum* and glucose in *N. laevis* resulted in higher Fx content compared to photo-autotrophic growth [76, 77].

### Challenges and future prospects

Despite numerous advantages like species diversity, trophic flexibility, availability of strains with fully sequenced genomes and unique valuable natural products like fucoxanthin, diatoms are still grossly underrepresented in commercial production systems compared to green algae and cyanobacteria. Till date hand full of species like *Pheodactylum tricornutum*, *Cylindrotheca* sp., *N. laevis* and *N. alba* are explored for commercialization. This misrepresentation might be due to their limited productivity due to issues related to limited understanding on optimal growth conditions under varying physicochemical parameters, contamination, and operational costs in present photo-autotrophic cultivation systems. With recent advances in diatom mixotrophic and heterotrophic growth, strain selection and development has led to greater emphasis on exploring them for commercial applications. But still major challenges like bioprospecting for new species which can grow under heterotrophic conditions, better understanding of their carbon metabolism, selective extraction of multiple functional biomolecules, development of large-scale culture systems for their sustainable production needs further exploration.

Lately, the potential of diatoms to cater as an ideal microbial cell factory synthesizing high value products has increased its scope of exploration. Though the two-stage cultivation processes and simulated nutrient depletion studies have shown a cooperative influence of carbon sources on biomass and lipid yields [78], concrete optimization and broad scale implementation of these practices is the major stumbling block. Extensive screening of multiple diatom species under adaptive conditions of mixotrophic and heterotrophic growth while utilizing alternative substrates with little or no pretreatment is required for developing cost-effective industrial scale production systems. Since most of the studies have focused on laboratory scale experiments, validation of these parameters when moving from the small to large scale trials needs to be conducted to ensure efficient valorization of the algae [89]. This kind of upgradation is essential for optimizing both the upstream and downstream bioprocesses like extraction, purification, and enhancement of the metabolites [90].

Subsequently, deploying approaches involving alternative substrates and varying cultivation conditions can aid in exploring the strain niches and identifying generalist or specialist behavior. Besides this integration of adaptive evolution with metabolic analysis, gene expression assays, modelling and networking tools could not only elaborate our understanding of the intracellular and extracellular crosstalk but also provide evidence for the rate limiting steps in these processes which can be targeted thereon.

## Conclusions

Diatoms are significantly different from other algal classes with respect to their unique evolutionary, genetic, cellular, and metabolic competencies. These attributes contribute to their high adaptability and amenability which enables them to outperform other algae in terms of their growth and lipid production in small as well as in large scale cultivation systems. Furthermore, their ability to grow in both light and dark conditions using varied carbon sources has been reported to trigger production of novel biomolecules such as EPA, fucoxanthin, chrysolaminarin, phytosterols which have pharmaceutical, nutraceutical, food, feed, and fuel applications. However, exploring them for commercial applications requires strategic novel biotechnological approaches in optimization of cultivation parameters, sustainable fermentation systems and integration of modern and adaptive technologies for enhanced downstream processing.
